# Creation of 21st century anatomy facilities: designing facilities for integrated preclinical education in the Middle East

**DOI:** 10.1186/s12909-023-04361-7

**Published:** 2023-05-26

**Authors:** Dietrich E Lorke, John A. Rock, Robert Hernandez, David Graham, Natalie Keough, Daniël J. van Tonder

**Affiliations:** 1 Department of Anatomy and Cellular Biology, College of Medicine and Health Sciences, Khalifa University, P O Box 127788, Abu Dhabi, United Arab Emirates; 2 College of Medicine and Health Sciences, Khalifa University, Abu Dhabi, United Arab Emirates; 3 Department of Medicine, Kerkorian School of Medicine, University of Nevada, Las Vegas, United States of America; 4 Department of Medical Imaging and Radiology, College of Medicine and Health Sciences, Khalifa University, Abu Dhabi, United Arab Emirates; 5 College of Medicine, American University of Antigua, Antigua, Antigua and Barbuda; 6 Department of Anatomy, School of Medicine, Faculty of Health Sciences, University of Pretoria, Pretoria, South Africa

**Keywords:** Anatomage table, Anatomy laboratories, Anatomy teaching, Dissection, Medical education, Plastination, Prosection, Sectra, Ultrasound

## Abstract

**Background:**

The establishment of new anatomy facilities needs to accommodate a combination of modern teaching modalities that best align with evidence-based best teaching practices. This article describes the process in which our state-of-the-art anatomy laboratories were designed and implemented, and how these facilities support aspects of modern anatomy education.

**Methods:**

A list of best practices for anatomy education in a modern medical curriculum was summarized from the literature. To assess student satisfaction, a survey related to student perception of the anatomy facilities (5-point Likert scale) was conducted.

**Results:**

Our educational modalities include a broad range of teaching approaches. The Instructional Studio houses prosected and plastinated specimens, and cadaveric dissections are performed. Each of our three Dry Laboratories allow for active learning and interaction between small student groups. The Webinar Room acts as a conference room for departmental and online meetings, discussions with students, and dialogues with affiliated hospitals via the internet. The Imaging Center is equipped with a Sectra® medical educational platform, CAE Vimedix® Virtual Medical Imaging Ultrasound Training System, and Philipps Lumify® Ultrasound devices to train students to conduct and interpret sonographic images. Moreover, the Complete Anatomy® program is made available to all our students.

**Conclusion:**

The layout of our newly created Anatomy Facilities allows for all aspects of modern medical education mentioned in the literature. These educational modalities and teaching approaches are highly appreciated by our faculty and students. Moreover, these technologies allowed for a smooth transition from on-site anatomy teaching to online education during the COVID pandemic.

## Introduction

Khalifa University of Science and Technology (KU), a public research university located in Abu Dhabi, the capital of the United Arab Emirates, was founded in 2007 to nurture a knowledge-based economy. Initially encompassing the College of Engineering and the College of Arts and Sciences, it was decided in 2018 to establish a College of Medicine and Health Sciences (CMHS) to address the growing demand in the country of well-trained physicians and allied health workers. KU opted to create a four-year postgraduate Medical Degree (MD) program modeled after the US American system. As such, the CMHS accepted 30 students in 2019 as its inaugural student cohort to graduate in 2023. Subsequently, they accepted 34, 48, and 44 students in the following years. Part of the planning and development efforts was the creation of state-of-the-art anatomy laboratories providing appropriate facilities for a growing student cohort to eventually accommodate between 100 and 120 students per graduating year, as well as focus on interactive student-centered teaching of medical and Allied Health Science students, and for organizing Continuing Medical Education (CME) activities. Another important objective was to provide facilities for anatomical, biomedical, radiological, orthopedic, and surgical research. Education in anatomy must be appropriate to Middle Eastern culture without compromising the training of skilled medical doctors. When establishing our new Medical College, it was not feasible to establish a body donation program, and other sustainable cadaver resources had to be investigated. Moreover, establishing a body donor program is very costly and requires at least five years before universities can avail themselves of cadavers. Hence, we had to create an anatomy laboratory that reduces the number of outsourced body donations to a minimum and that can accommodate and implement current best practices in anatomy education.

Estai and Bunt (2016) summarized, in a critical review, that the current best practices to teach anatomy in a medical curriculum is to combine various teaching resources, e.g., plastic models, plastinated human specimens, computer-assisted learning modalities, dissection, prosected specimens, as well as ultrasound, radiographs, CT and MRI images [1]. This solidified the comments by Kerby et al. [2] who state that currently there is no single teaching methodology that satisfies all the learning outcomes of a medical curriculum. Therefore, the creation of our newly created Anatomy Facilities aimed to address all these aspects of modern medical education. This included the concepts of integration of anatomy with radiology, ultrasound imaging and clinical skills, the possibility of small group interactive self-directed studies, access to a variety of educational technologies, and exposure to dissection and prosected specimens. In recent years, the methodology of anatomy education has been transformed by new technologies, such as plastination [3–6], computer-assisted learning tools, e.g., the Anatomage® tables [7, 8], CAE Vimedix® ultrasound simulator mannequins [9, 10], Complete Anatomy® program [11], as well as the creation of affordable, high-quality plastic models [12–14].

At KU, anatomy is taught in an integrated system-based fashion. During period one of the curriculum (Foundations of Medicine), a four-week Introductory Anatomy Course, covering the basics from the microscopic to the organ system levels, is taught in the third month of the first year. This follows a course on genetics, biochemistry, and cellular biology, during which students are introduced to fundamental concepts of histology. During this introductory course, the basics of gross anatomy are taught in parallel with imaging technologies and radiological anatomy. In addition, case-based learning sessions introduce the students to clinical scenarios, during which they apply their knowledge in anatomy and clinical skills to solve diagnostic and therapeutic problems. Out of the 56 teaching hours of the first-year introductory course, there are 36 interactive lectures, using TurningPoint (©2021, Turning Technologies®, Youngstown, Ohio, USA), covering basics of embryology (six hours), histology (four hours), radiology (six hours) and gross anatomy (20 h). During these lectures, using Turning Point and other audience response systems, students are regularly presented questions (about 2–4 times per one lecture hour), and subsequently the answers are discussed, and misconceptions addressed. In addition, we also use an interactive approach in the dry labs (see below). The interactive lectures are complemented by twelve hours in the laboratories, five hours of case-based-learning and three hours of tutorial exercises. During the twelve-month-long second period of the curriculum (organ-systems), the anatomy of the individual organ systems is taught in greater detail in the context of clinical case scenarios, integrated with radiological anatomy, pathophysiology, therapeutics, and clinical skills. Depending on the individual course, 15–50% of the curricular activities of period two courses cover anatomy.

Since plastic models are shown to be efficient supplements in teaching anatomy [12–14], our first period medical students start their gross anatomy laboratory sessions with plastic models (©2021, SOMSO®; Marcus Sommer Modelle GmbH, Coburg, Germany). In a study comparing learning outcomes of students using plastic models with students studying from cadaveric prosection to learn musculoskeletal anatomy of the upper limb, no significant difference in students’ performance was observed between the two groups [14]. This is supplemented by the Complete Anatomy® program (©2021, 3D4Medical®, Elsevier, Dublin, Ireland), which is made available to all students at the beginning of their studies. Plastinated specimens [4–6] are also employed during the laboratory sessions to give students a more realistic impression of anatomical relationships. In parallel, students also get accustomed to the Anatomage® virtual dissection table (©2021, Anatomage Inc. Santa Clara, CA, USA), which is used during the laboratory sessions as an additional resource. During the second half of the Introductory Anatomy Course, they are also introduced to prosected specimens of the thorax and abdomen. During the integrated organ system courses of period two, all these teaching resources are employed during laboratory sessions, which are based on clinical case scenarios and related problems that the students must solve in study groups. These approaches are focused on active and peer-discussion learning. Additionally, ultrasound modalities and the Sectra® table (©2021, Sectra AB, Linköping, Sweden) are utilized as additional educational tools to teach anatomy in a clinical context for contextualized learning. During period four of the curriculum (Advanced Clinical Rotations), motivated students can choose to participate in an “anatomy dissection elective”, during which a group of four to six students performs dissections on embalmed human cadavers. The prosected specimens prepared during this course are utilized in period one and two courses.

The requirements for the construction of our anatomy facilities were therefore to create educational spaces that promote teacher-learner interaction, peer-assisted learning, and self-directed studies. The motivation of this project stems from the need to address specific problems when establishing anatomy facilities that allow for a combination of multiple pedagogical resources. This manuscript is beneficial for providing insight into processes and considerations for establishing Anatomical Facilities aligned with current evidence-based research.

## Materials and methods

During our literature search, we were guided by the “Preferred Reporting Items for Systematic reviews and Meta-Analyses” (PRISMA) statement [15, 16]. Using the PubMed, Scopus and EMBASE databases from January 1, 1990, to October 31, 2022, we included the search terms “anatomy”, “laboratory”, “medical education”, “preclinical education” “evidence-based anatomy education”. Subsequently, further searches were performed, including key words “plastination”, “3D models”, “Anatomage”, “Sectra”, “CAE Vimedix”, “dissection”, “prosection”, “ultrasound”, “sonography”, “augmented reality” and “virtual reality”. Citations within literature were also reviewed for relevant articles. Included were publications written in English, German, French and Spanish.

In 2015, Brenner and colleagues [17] proposed six techniques for anatomy education, which included in-person lectures, cadaver dissection, inspection of prosected material, models, living and radiological anatomy teaching, and computer-based learning (VR, AR, and 3D) [18]. Suggestions for optimal modern curricula were also established by the Education Committee of the Anatomical Society of Great Britain and Ireland (ASGBI) and published in 2007 [19], which emphasize that maximum learning can be achieved using the following: (1) dissection/prosection, (2) multimedia, (3) practical procedures, (4) surface and clinical anatomy, and (5) radiological imaging.

In this section we followed the systematic approach of a type of “needs analysis” by constructing a list of instructional methods and their effectiveness, based on the above prescribed “best teaching practices”, and from the authors experience (Table 1). This represented the educational “needs” for delivering quality anatomy education. Following this, a list of facilities required to achieve these best practices (needs) was identified and was purposed with setting the foundation for the construction of a compliant facility in which to teach anatomy to modern medical students in the UAE (Table 1).

**Table 1:** List of considered “best instructional methods” with aligned facilities/resources required

Best method	Evidence of effectiveness:	Facilities or resources required:
**Instructional method 1: Dissection (body donors)**	Teaches self, active, and deep learning skills; prepares students for clinical practice, for encounters with death; practice of manual skills; understand relationship between patient symptoms and pathology [38–41]. Develops medical professionalism (teamwork competency), stress coping mechanisms, and empathy [42]. Exposure to anatomical variations [43]. Enhances student learning experience and content retention as an extracurricular activity [44]	Body donor program OR access to outsourced body donors. For inhouse body donor program → embalming, maceration, and disposal facilities. Storage (body donor fridges, cold room). Dissection laboratory (adequate space and ventilation). Dissection equipment (dissection guides; dissection instruments).
**Instructional method 2: Prosection (body donors; prepared specimens)**	Is flexible, contextual, and time efficient; fewer body donors are needed [45, 46]. Allows to identify and view more anatomical structures and possible variations than during dissection [47]. Using prosection is more than adequate to aid anatomy learning [40]. Is suitable for time constrained curriculum (no need to dissect).	Storage facilities (fridges; cold room) Body donors to prepare specimens from (own body donor program OR access to outsourced body donors). Suitable wet laboratory space for demonstrations (adequate ventilation). Anatomical atlas.
**Instructional method 3: Plastinated specimens and/or plastic models**	Are odorless, convenient to store, and easy to handle [41, 48, 49]. Enhance students’ appreciation and use of plastinated specimens; enhance knowledge content [48]. Aid in realistic visualization of anatomical concepts often difficult to describe or see in student-driven dissection [50]. Suitable for time-constrained curricula (no need to dissect). Expose anatomical structures not always visible during traditional dissection	Adequate storage (protect specimens). Suitable laboratory space for demonstration. Anatomy atlas.
**Instructional method 4: Multimedia (computer-based learning; VR; AR; MR; drawing)**	Increase motivation to learn and knowledge retention [51, 52]. Enhance interaction with content, increase subject interest, and motivation to learn [53, 54]. Increase student test scores with use of AR/VR/MR as a supplement [55, 56]. Drawing increases understanding of complex material; helps instructor identify gaps in learner knowledge [57, 58]	Adequate and dedicated laboratory space (virtual lab). Adequate computer resources (Complete anatomy, Primal pictures, Clinical Key, HoloLens with CAE Vimedex Software). Dedicated drawing areas (special wall paint for drawing, drawing boards). Access to laptop/tablet for students.
**Instructional method 5: Living anatomy (surface anatomy)**	Allows palpation and auscultation of living individuals who can be asked to change position [59]. It speaks to “Peer Physical Exam” (PPE) which involves students physically examining each other and has been found to be more useful than models [60]. Provides students with opportunity to safely practice and master clinical skills prior to patient encounter as well as development of empathy and improving communication skills [61, 62]	Adequate lab space. Examination tables. Plastic models for correlation.
**Instructional method 6: Medical imaging (x-ray; CT; MRI; ultrasound)**	Permits in vivo visualization of body structures and possible pathology in two dimensions and enables learners to gain anatomical knowledge in a clinically relevant context [63] Integrates imaging techniques into medical curricula [64], which is considered a valuable addition to dissection-based instruction, as it promotes better understanding of anatomical spatial relationships [65, 66]. Provides valuable addition to dissection-based instruction [66–68].	Dedicated imaging centre (high quality computer screens). Imaging resources (Sectra). Ultrasound devices (hand-held, mannequins).

### Student survey

To assess student satisfaction with the current facilities and resources, period one and period two students (79 participants altogether) were given a survey (Institutional Review Board Protocol #H21-041). The survey, which adhered to the “Strengthening the Reporting of Observational studies in Epidemiology” (STROBE) guidelines [20, 21], consisted of seven questions formulated to assess how the students benefited from the instructional methods mentioned in Table 1. These questions were composed using guidelines set out by Nemoto and Beglar [22] in combination with procedures used by Spooren et al. [23].

Table 2 depicts how these questions were formulated to align with requirements to assess student satisfaction perceived by “best practice” instructional methods. For each item, students were asked their agreement with a statement on a 5-point Likert scale (1 = strongly disagree; 2 = disagree; 3 = neither agree nor disagree; 4 = agree; 5 = strongly agree). In addition, students were asked to provide open comments related to their experiences. Cronbach’s coefficient (α) was used to calculate the internal consistency coefficients of the items included in the survey [24].

**Table 2:** Formulated questions to align with requirements to assess student satisfaction perceived by “best practice” instructional methods (see Table 1)

Question	Instructional methods
I believe that the laboratory session fostered my understanding of the course content	1, 2, 3, 4, 5, and 6
Plastic models are appropriate and very useful tools during the initial anatomy teaching as a first approach to understanding the structure of the human body.	3
The use of Complete Anatomy as part of a combination of teaching tools positively influenced my acquisition of gross anatomical knowledge.	4
I am more satisfied with a combination of plastic models, plastinated specimens, prosected specimens, and computer assisted technology than with only one of these teaching modalities alone.	2, 3, 4 and 6
I prefer cadaveric prosection and dissection, compared to plastic, plastinated and computer models, when asked how to best understand anatomical relations and to gain anatomical knowledge in a clinical context.	1, 2
Integration with clinical cases helped me understand the importance of anatomy.	5
Combination of prosected and plastinated specimens, together with the Complete Anatomy program, fosters my anatomy knowledge better than each of these teaching methods alone.	2, 3 and 4
“Open question” Please comment on how you would improve the laboratory session regarding the plastinated, plastic models, prosected specimens, and virtual anatomy content.	2, 3, 4 and 6

## Results

**Fig. 1:**
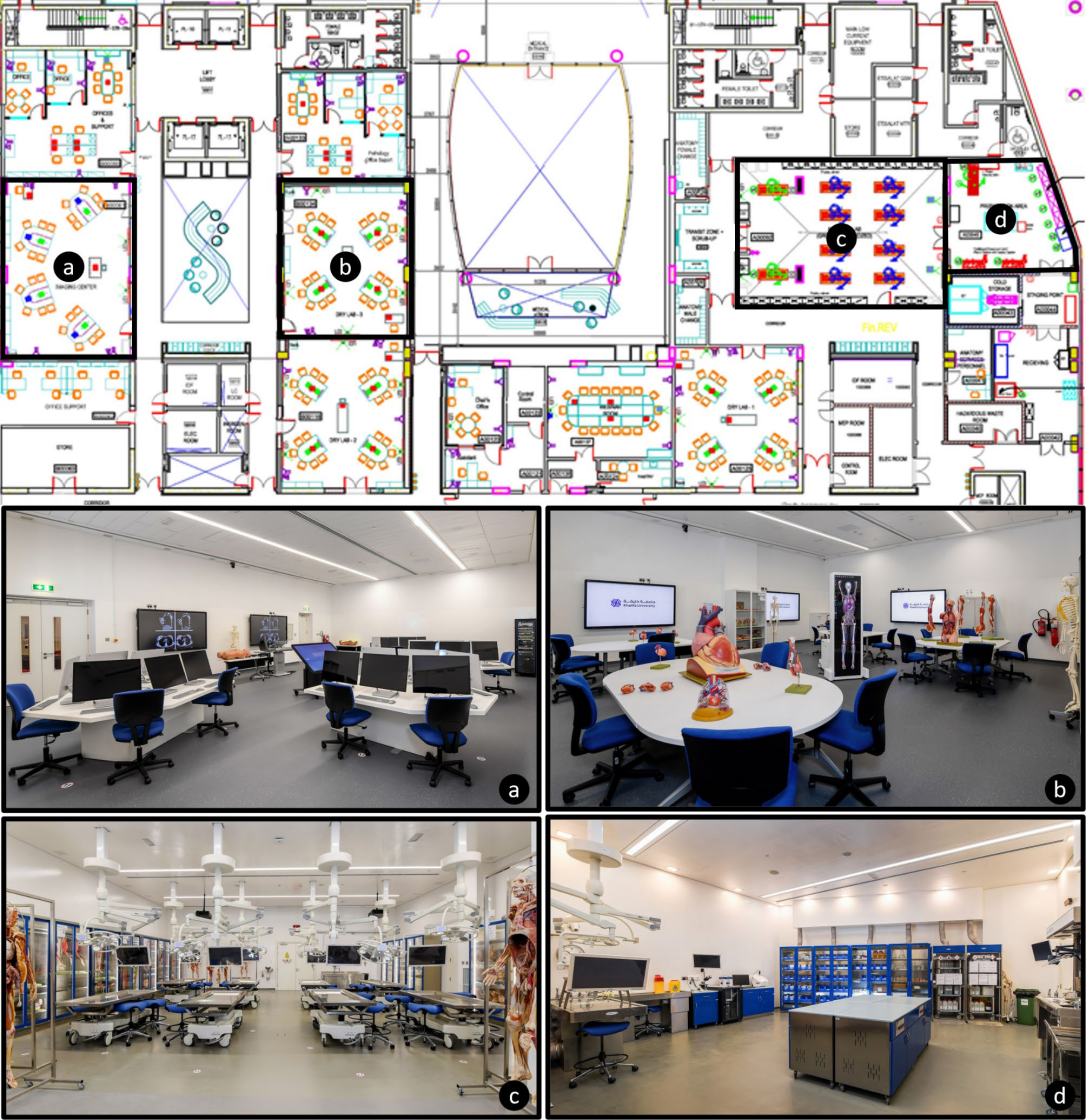
Top. Floor plan of Buildings A and B on the ground floor of the College of Medicine, Khalifa University. Overview of **a**. Imaging Center; **b**. One of the three Dry Laboratories; **c**. Instructional Studio; **d**. Preparation Room

Based on the criteria set out in the material and methods, i.e., prosection, plastinated specimen, cadaver-based dissection, medical imaging, living anatomy and multimedia, the design of the facilities in the Department of Anatomy was aligned to meet with these expectations. The following facilities (Fig. 1) were planned and constructed to align with the requirements to achieve the “best practice” instructional methods (Table 1). Each of the facilities and how they integrate with the teaching approaches is detailed in Table 3.

**Table 3:** Facilities planned and constructed to align with requirements to achieve “best practice” instructional methods (see Table 1)

Facilities (Quantity)	Instructional methods
Dry Laboratory (three)	3, 4, 5, and 6
Instructional Studio – Wet Laboratory (one)	1 and 2
Imaging Center (one)	4, 5, and 6
Webinar Room (one)	4
Preparation area (one)	Required to prepare for instructional methods 1 and 2
Refrigerator and Freezer Rooms (two)	Required for storage of specimens used for instructional methods 1 and 2

### Dry laboratories

**Fig. 2:**
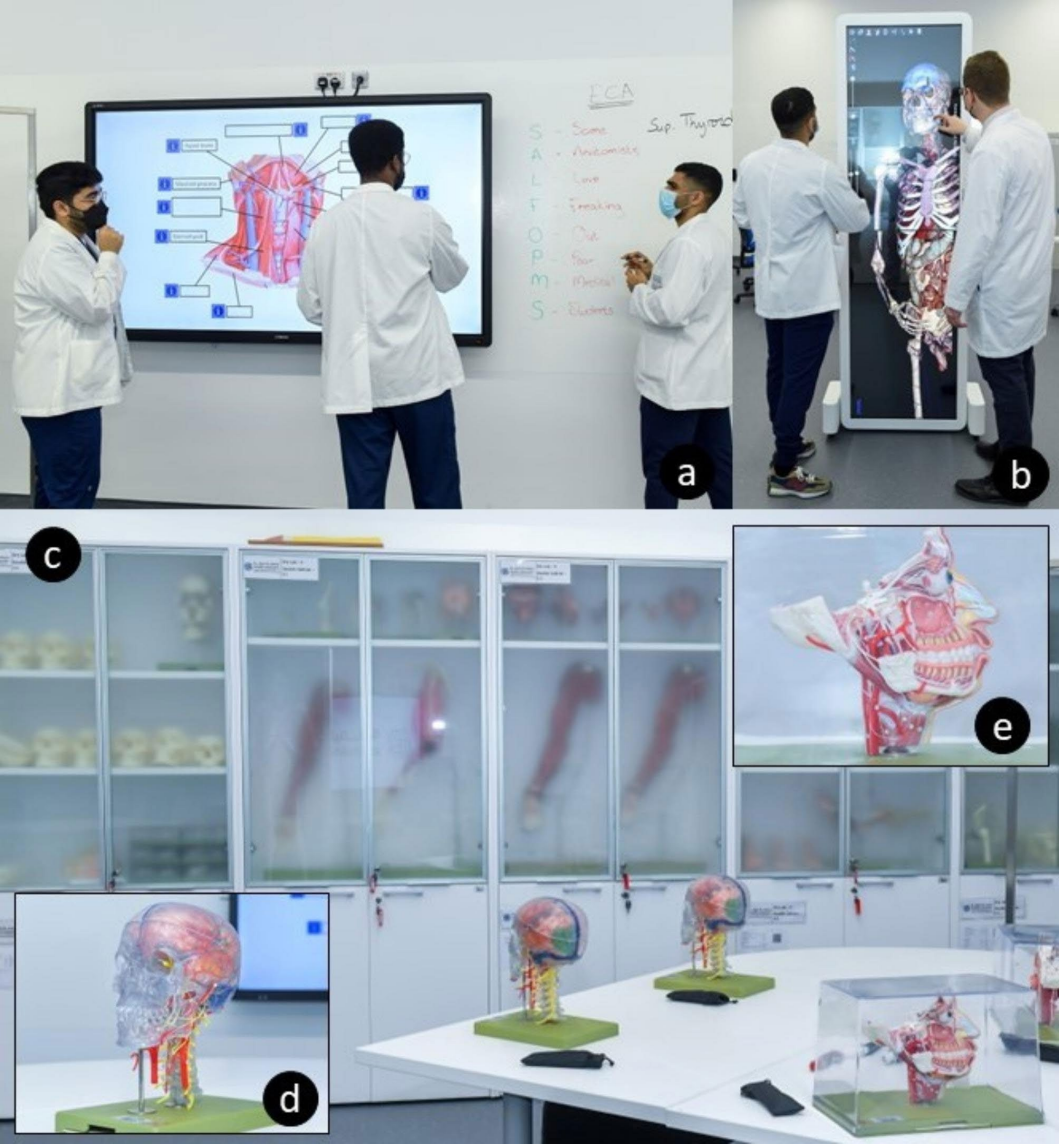
Dry Laboratory with: **a**. Student guided teaching session in the Dry Laboratory utilizing the CTOUCH® 85” Laser Nova UHD interactive screens and the gloss painted walls, providing an active learning environment. **b**. The Anatomage® virtual dissection table used in a vertical position, aiding students in 3D anatomy visualization. **c**. Typical Dry Laboratory demonstration session; relevant SOMSO® models are displayed to aid in student teaching. **d**. SOMSO® model which can be disassembled by removing the cranium and inspecting the removable brain contained within. **e**. Highly detailed SOMSO® model to illustrate the intricacies of the infratemporal fossa, nasal cavities, and mandible

Each of the three Dry Laboratories caters for 24 students and one lab instructor. In addition, audio-visual support is continually provided by the KU IT support office. The Dry Laboratories are designed and fully equipped to deliver diverse teaching methodologies, with the ultimate focus on student-centered learning and facilitation. This includes five CTOUCH® 85” Laser Nova UHD (©2021, CTOUCH, Eindhoven, Netherlands) interactive screens that broaden the capabilities of teaching and presenting content to both small and large student groups during dedicated laboratory sessions (Fig. 2.a.). Students collectively work in four groups of five to six students on a clinical scenario or a functional anatomical problem. Subsequently, the results of their discussions are presented on their respective screens and discussed with the other participating groups. The laboratories are centrally controlled with the Creston® application (©2021, Crestron Electronics, Inc., Rockleigh, New Jersey), allowing the venue to function both as a local, in-person presentation site, as well as a distance, off-campus learning site due to the live streaming capabilities captured with fixed and mobile Q-SYS (©2023, QSC, LLC, Costa Mesa, California, USA) PTZ-IP conference cameras and integrated Extron (©2023, Extron Electronics, Anaheim, California, USA) SMP 300 Series sound solution for capturing and distributing AV from handheld Shure (©2023, Shure, Niles, Illinois, USA) microphones and Shure ceiling array microphones. With this application, the instructor can project content from several resources within the venue, such as from the main podium, a secondary input device, a Clickshare® (©2023, Barco, Kortrijk, Belgium) device, and an Anatomage® table, onto the five interactive screens. The system also has the capability to mirror the content of one of the five CTOUCH® interactive screens to any or all the available screens. This sharing capability is also possible across the three Dry Laboratories, allowing content presented in one laboratory to be shared with and projected into the other two laboratories simultaneously.

Situated within each Dry Laboratory is an Anatomage® table, which provides students with a virtual dissection tool for anatomy education and aids them with three-dimensional visualization of anatomical relations. The Anatomage® table can be orientated both in a horizontal or vertical position, providing a different perspective for the students to interact with the table (Fig. 2.b.). The Anatomage® table not only provides a virtual component for dissection and prosected specimens, but also enables the students to visually correlate the imaging modalities (MRI, CT scan) with the virtual specimens, thereby linking anatomy with radiology and clinical applications.

The Dry Laboratories also house a wide variety of plastic SOMSO® anatomical models (Fig. 2.c.). The large collection of SOMSO® models has been meticulously selected to accommodate and represent all the relevant anatomy taught across the courses offered in the integrated medical curriculum. A large proportion of the models has been selected based on their ability to be disassembled and reassembled (Fig. 2.d), a unique feature that allows students to study and appreciate the complex nature and the relations of the human body (Fig. 2.e) in a systematic and fun way. The SOMSO models were thoroughly compared to other providers of models with the same technical specifications and were, in most cases, selected above their counterparts for their anatomical accuracy and build quality.

Additionally, sections of the laboratory walls are painted with high gloss paint, which can be used as a whiteboard (Fig. 2.a.), thus aiding in the process of active learning and giving the opportunity for faculty and students to illustrate complex anatomical concepts in a visual manner, while discussing the content at hand. The Complete Anatomy® program is also available for our students and was selected as the preferred application, following a comparison with several similar applications, for its user-friendly interface, quality 3D renders, and anatomical accuracy.

### Imaging center

The Imaging Center, located opposite the Dry Laboratories, has 24 student stations and one instructor station, each setup with Microsoft Surface Studio 2® (©2023, Microsoft Corporation, Redmond, Washington, USA). This unique setup allows faculty and students to view medical images on high quality screens with various DICOM viewers. The instructor can project the content from the instructor screen to all 24 student stations, as well as to the two CTOUCH® 85” Laser Nova UHD interactive displays. This ability to live share allows the instructor to teach, explain and demonstrate fine anatomical features and abnormalities on radiographs, CTs, or MRIs from a central point. This feature has been most useful during the social distance restrictions brought on by the COVID-19 pandemic.

The Imaging Center also houses a Sectra® table, which is a multi-touch display workstation that enables faculty and students to access the Sectra Education Portal®. This permits engaging group lessons and lectures within the Imaging Center. The Sectra Education Portal® allows high-quality DICOM cases, curated by collaborating universities, to be viewed and permits tactile alterations to accommodate for the requirements of individual modules taught in the medical school. In addition to the Sectra Education Portal®, the Sectra® table includes VH Dissector Pro (©2023, Touch of Life Technologies Inc, Aurora, Colorado, USA) that offers high quality 3D reconstructed images, together with cross sectional views to easily disassemble and identify anatomical structures.

With the increasing use of and push to incorporate more ultrasound teaching into medical curricula, the Imaging Center is supplemented with two CAE Vimedix® ultrasound simulator mannequins. One male mannequin for transthoracic, transesophageal echocardiography (©2023, CAE Vimedix Cardiac, CAE Healtcare®, Montreal, Canada) and a second female OB-GYN mannequin (©2023, CAE Vimedix OB-GYN, CAE Healtcare®, Montreal, Canada) to perform obstetric and gynecological transabdominal and endovaginal ultrasound examinations. Accompanying these mannequins is a comprehensive user interface that enables the instructor to present both imaging representative of normal anatomy and of pathologies of multiple diseases to support specific course objectives. The CAE Vimedix® can communicate with a Microsoft Hololens 2® (©2023, Microsoft Corporation, Redmond, Washington, USA), which allows for the 3D content to be projected onto the mannequin with augmented reality (AR) images. To fully expand and integrate this area with the rest of the laboratory spaces, the Imaging Center has the same presentation and sharing abilities as the Dry Laboratories, with two CTOUCH® 85” Laser Nova UHD interactive screens. In addition, 20 Philips Lumify® (©2023, Koninklijke Philips N.V., Amsterdam, Netherlands) ultrasound devices are regularly available to students to experience the complexity of explorative anatomy on themselves and standardized patients.

### Instructional studio

The Instructional Studio was designed and equipped to fulfill two main functions. Firstly, to serve as an integrated teaching facility to medical students and secondly, to serve as a sophisticated venue to run CME courses to external stakeholders. The studio is fitted with standard STARLED5 NX (©2021, ACEM Spa, Argelato, Bologna, Italy) surgical lighting, VarioView 32 (Ondal Medical Systems GmbH, Hünfeld, Germany) adjustable screens, and AXCam.FHD (©2021, ACEM Spa, Argelato, Bologna, Italy) cameras at each of its eight student and two instructional stations. This intuitive setup allows for the integrative Xe® system (©2018, TechLab Works s.r.l, Blacksburg, VA, USA) software control system to remotely control the content projected on any of the station screens, including the two QM85F Samsung® Color Display Unit (©2021, Samsung, Suwon-si, South Korea) screens and two REALiS 4K501ST Pro AV (©2021, Canon Ota City, Tokyo, Japan) 4 K projectors situated on either side of the venue. In addition to the video routing function, the content visible on the respective camera can be recorded and streamed.

**Fig. 3:**
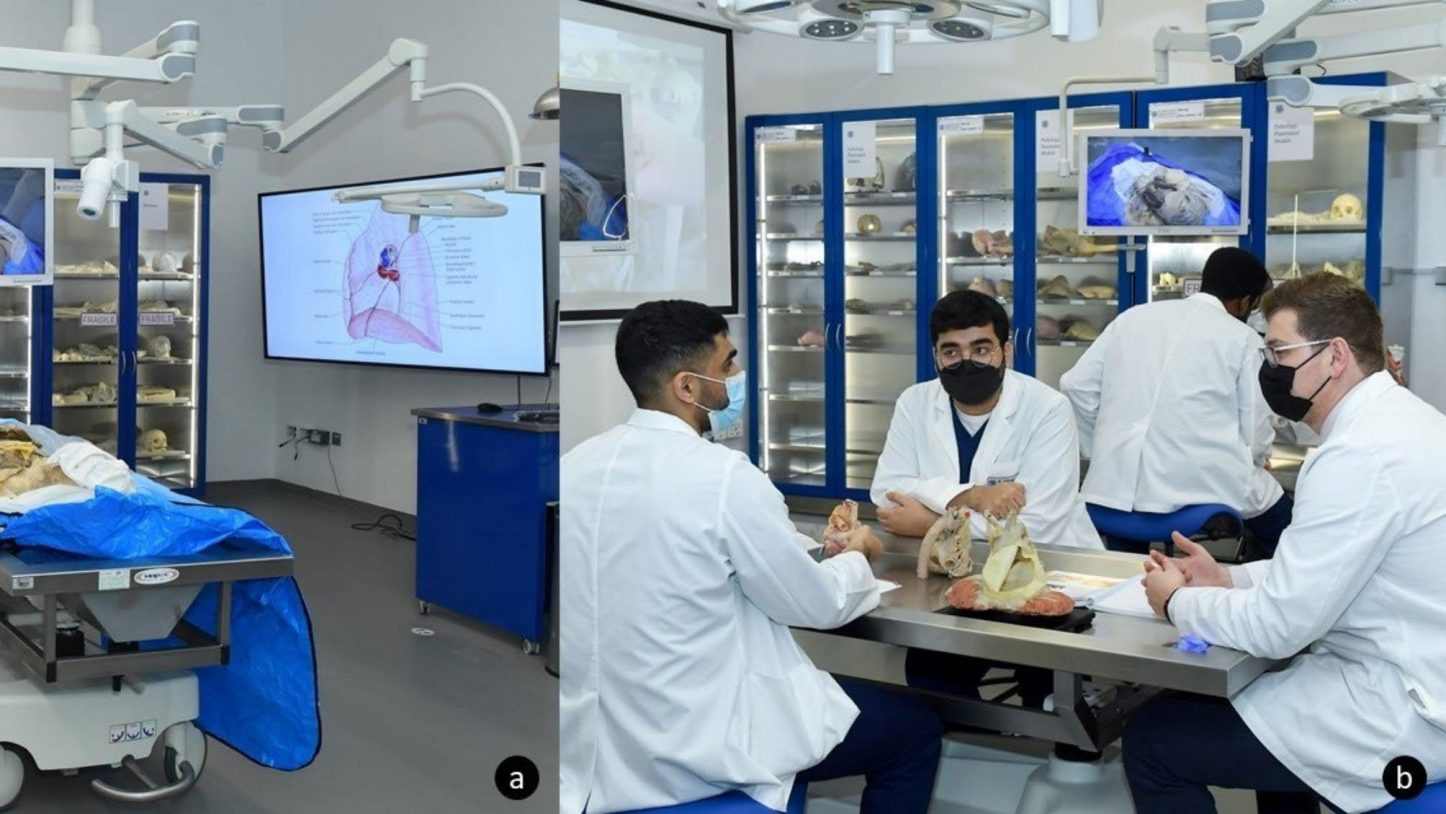
Instructional Studio with: **a**. Mobile Mopec® Hydraulic Lift Dissection Table and attached ventilation system; **b**. Period two teaching session using von Hagens Plastination® specimens

The two instructional stations are equipped with individualized ventilation to prevent evaporation of fumes into the laboratory when dissection of embalmed specimens is conducted. These instructional stations are deliberately mobile with Mopec® Hydraulic Lift Dissection Tables (©2021, Mopec, Madison Heights, MI, USA) to allow space and mobility for the dissectors while dissecting (Fig. 3.a.). Along the walls of the Instructional Studio, there are both plastic SOMSO® models and von Hagens Plastination® (©2023, Gubener Plastinate GmbH, Guben, Germany) specimens stored in easily accessible and protective cabinets, to aid in teaching during sessions scheduled in the venue (Fig. 3.b.). Currently KU houses one of the largest collections of von Hagens Plastination® specimens outside Gubben, Germany, with more than 160 specimens. This collection includes various stages of dissection of six full bodies, 20 head and neck specimens, ten brains, nine pelvis and perineum dissections, four thorax specimens, 13 upper limbs, 21 lower limbs, and 46 organs relating to the cardiovascular, respiratory, gastrointestinal, and urinary systems. In addition, 29 pathology specimens are available, which range from pathologies such as liver cirrhosis to examples of smokers’ lungs. Moreover, the collection includes two fully disarticulated skeletons, and two complete central and peripheral nervous systems.

### Preparation room

Adjacent to the Instructional Studio is the Preparation Room, which has a right-handed L-shaped elevating autopsy table with an integral wing dissection table (CE650, Mopec, Madison Heights, MI, USA) with standard STARLED5 NX® surgical lighting, VarioView 32® adjustable screens, and AXCam.FHD® camera capabilities linked to the Instructional Studio display system via the Xe® system. This allows sensitive dissections to be conducted and projected to students not physically present within the room. Again, these specific and tailored setup mechanisms have allowed anatomy teaching to continue amidst the restrictions imposed by the COVID-19 pandemic. This room was also designed to allow for preparing prosected specimens from donated cadavers; it has the storage capacity to keep these prosected specimens until needed. The Preparation Room also contains, to complement the histology capabilities, four ultra-cold surface units (histology freeze plates). These units can freeze histology samples quickly, thereby increasing curing process of tissue blocks, allowing dissection of frozen tissues, and reducing the overall tissue processing time by around 40%. Histology freeze plates are a necessary component for microtome sectioning in the absence of cryostat.

Accompanying the dissection table and cold surface units, are two Mopec® Maestro Grossing Station® (©2021, Mopec, Madison Heights, MI, USA) and one 3DHistech Pannoramic Midi II® (©2021, 3DHISTECH Ltd., Budapest, Hungary) histological scanner. The equipment is connected to Mopec®’s PathCam Gross Imaging System (©2021, Mopec, Madison Heights, MI, USA) to simplify tagging specimens and histological microscopy. As each of the two grossing stations is equipped with a camera and accompanying software to identify barcodes, this works in harmony with the 3DHistech Pannoramic Midi II®, which has the same capabilities. The software SlideCenter® (©2021, 3DHISTECH Ltd., Budapest, Hungary) provided by 3DHistech® permits specimens to be examined remotely while a laboratory technician works on the cadaveric samples within the laboratory.

### Webinar room

The Webinar Room comfortably seats 18 participants to engage in the exchange of ideas between faculty, staff, students, and external stakeholders. It provides multiple avenues to interact and involve various members of any discussion with a CTOUCH® 85” Laser Nova UHD interactive screen and an Epson® EB-710Ui Ultra Short Throw Projector (©2021, Seiko Epson Corporation, Suwa, Nagano, Japan) connected to an Epson® H599 LCU Projector Touch Unit (©2021, Seiko Epson Corporation, Suwa, Nagano, Japan) on either side of the venue. In addition, scattered around the oval-shaped conference table are four cubbies, which present the opportunity for any participant to project the content to any available displays, either with a HDMI, VGA, Ethernet, or a USB connection utilizing the Clickshare® functionality. Together with the Creston® integration, this room can stream the proceedings over the internet or project videos and audio to any of the Dry Laboratories and to the Imaging Center with the help of fixed and mobile Q-SYS PTZ-IP conference cameras and integrated Extron SMP 300 Series sound solution for capturing and distributing AV from handheld Shure microphones and Shure ceiling array microphones, establishing the interconnected ecosystem to relay content to any desired location.

### Student survey: results

Student reactions towards the anatomy modalities (Table 4) were positive as the response median was 5 (strongly agree) for six of the questions, which included: “I believe that the laboratory session fostered my understanding of the course content”, “Plastic models are appropriate and very useful tools during the initial anatomy teaching as a first approach to understanding the structure of the human body.”, “The use of Complete Anatomy as part of a combination of teaching tools positively influenced my acquisition of gross anatomical knowledge.”, “I am more satisfied with a combination of plastic models, plastinated specimens, prosected specimens, and computer assisted technology than with only one of these teaching modalities alone.”, “I prefer cadaveric prosection and dissection, compared to plastic, plastinated and computer models, when asked how to best understand anatomical relations and to gain anatomical knowledge in a clinical context.”, “Integration with clinical cases helped me understand the importance of anatomy.” For just one question, the median was 4 (agree) out of 5: “Combination of prosected and plastinated specimens, together with the Complete Anatomy program, fosters my anatomy knowledge better than each of these teaching methods alone.”

**Table 4:** Results of questions regarding student satisfaction perceived by “best practice” instructional methods

Question	Score (Median)
I believe that the laboratory session fostered my understanding of the course content	5
Plastic models are appropriate and very useful tools during the initial anatomy teaching as a first approach to understanding the structure of the human body.	5
The use of Complete Anatomy as part of a combination of teaching tools positively influenced my acquisition of gross anatomical knowledge.	5
I am more satisfied with a combination of plastic models, plastinated specimens, prosected specimens, and computer assisted technology than with only one of these teaching modalities alone.	5
I prefer cadaveric prosection and dissection, compared to plastic, plastinated and computer models, when asked how to best understand anatomical relations and to gain anatomical knowledge in a clinical context.	5
Integration with clinical cases helped me understand the importance of anatomy.	5
Combination of prosected and plastinated specimens, together with the Complete Anatomy program, fosters my anatomy knowledge better than each of these teaching methods alone.	4

Scores were evaluated on the 5-point Likert scale and are represented as medians.

The survey consisted of 7 items and the value for Cronbach’s Alpha for the survey was α = 0.693, which is the minimally acceptable value.

## Discussion

This paper provides a clear guideline of how best practices for modern anatomy education listed in the Methods section (Tables 1 and 3) were meticulously considered and implemented with the design and layout of our newly created Anatomy Facilities. The design and implementation of these facilities and resources had to create a learning environment promoting student-centered learning, peer discussions and group interactions. The outcome was positively received by our students when factors such as using cadaver-based dissection, use of plastic and/or plastinated models, integrated imaging techniques, and multimedia supplementation were considered.

Research has shown that ***plastic models*** are a useful tool to teach anatomy, because they are easily accessible, relatively affordable, and very effective in promoting learners’ level of gross anatomical knowledge [13]. They allow students to obtain a notion of the three-dimensional arrangements of anatomical structures by rotating the organs in their hands and often by opening them up to investigate their interior. They have been shown to be particularly useful in teaching spatial brain anatomy [25]. According to our survey, plastic models are appropriate and very useful tools during the initial anatomy teaching as a first approach to understanding the structure of the human body.

The advantages and disadvantages of ***dissection***, the “systematic exploration of a preserved human cadaver by sequential division of tissue layers and the liberation of certain structures … with the aim of supporting the learning of gross anatomy” [26] have been discussed in detail [27]. Essentially, the emotional impact of dissection, health and safety issues, practicalities, and the cost of using cadavers have been mentioned as disadvantages. Potential advantages are better knowledge acquisition and integration, appreciation of three-dimensional relationships and anatomical variability, peer-group learning, tactile experience, promotion of professionalism through direct encounter with the cadaver, and development of manual skills required for many medical specialties [27]. A systematic review of the relevant literature dealing with objective data evaluating the cognitive learning outcomes of cadaver dissection compared to other teaching approaches concluded that there is a slight advantage of dissection over prosection [26]. Generally, the term “dissection” is employed when students are actively dissecting, whereas “prosection” refers to the study of cadaver specimens prepared by others [26]. We developed and offered a “Clinically Orientated Anatomy Dissection” course as an elective in 2022, in which eight Period 4 students enrolled and successfully participated. We believe that the quality of dissection performed by these students was well above standard compared to first year dissections seen in other medical curricula, as these students were more aware of the clinical significance and focused on preserving anatomical structures rather than conducting exploratory dissection. Additionally, we provide the opportunity for Period 1, 2, and 3 students to dissect weekly during a student interest group activity. Over the past 3 years, the average student attendance for this student interest group activity ranged between 10 and 18 students, depending on their curricular responsibilities, bringing the number of students participating in dissection to about a third of our student cohort. According to our own survey, which is supported by the literature [28], students generally prefer cadaveric prosection and dissection, compared to plastic, plastinated and computer models, when asked how to best understand anatomical relations and to gain anatomical knowledge in a clinical context. For the reasons listed above, we decided to include prosected specimens in our anatomy teaching and to allow motivated students to perform cadaveric dissection, but to reduce the number of body donations to a minimum and to supplement dissection with other teaching modalities.

Many of the advantages of cadaver prosected specimens, (e.g., tactile experience, three dimensionality and exact representation of the human anatomy), without the disadvantages, (e.g., problems in procurement, difficulties in preservation, large infrastructural requirements for storage, lack of resistance to mechanical strain, exposure to fixatives, smell, and easy destruction), can be achieved by ***plastination***. This method of preservation of organic material produces long-lasting anatomical specimens of body parts or the entire body [4–6]. During the process of plastination, the body fluids of dissected organs, organ systems or entire bodies of body donors are substituted with acetone, and subsequently, acetone is replaced by a polymer, resulting in clean, dry, odorless, non-toxic, touchable, durable and authentic specimens representing the exact anatomy of the body donor that can be easily transported [4, 5]. Moreover, thin plastinated organ slices impart a sound knowledge of cross-sectional anatomy, which is invaluable for the understanding of radiological images [29]. Similar sentiment is held by our students with comments in the survey suggesting “… Plastinates models gave a good idea of the reality and in visualizing …” and “I would like to use more the plastinated models because they are a more real representation of anatomy…”. The initial purchase is relatively costly, but due to the durability of the specimens lasting over decades [5], the investment pays off in a relatively short period. KU sourced its plastinated specimens solely from von Hagens Plastination® after an ethical sourcing inquiry. The authors personally visited the Plastination facilities and went through the records to ensure that plastinated specimens of interest were donated with the highest of ethical standards, including written consent. In addition, the tenet of body donation from von Hagens Plastination® is stipulated as follows: “Body donation for Plastination to the Institute for Plastination is not a contract, but a declaration of intent. The donor declares during their lifetime that their body should not be buried after death, but rather transferred to the Institute for Plastination. The consent form and a 30-page brochure provide detailed information so that each donor is explicitly informed about the future use of their body, including the production of teaching specimens for sale. The Institute for Plastination also organizes regular meetings for body donors. To ensure that the decision to donate one’s body to the Institute for Plastination is undertaken with free will there is no financial compensation.”

Given the time-constraints of modern anatomy curricula, the use of plastinated specimens also offers the opportunity to utilize teaching time more efficiently. During a standard three-hour dissection session, half the time is spent cleaning areas of interest (i.e., removing fat and fascia), during which no anatomy is taught. By using plastinated specimens, the relationships of anatomical structures are maintained and to some degree even clearer than those often seen in cadavers [30]. Specific areas that, due to time restraints, are not often seen in the dissection hall may be perfectly visible on the plastinated specimens due to their high quality of dissection. This environment allows students to appreciate the accurate anatomy and, importantly, the anatomical relations and integrate these with carefully prepared clinical scenarios and case-based discussions. For this reason, we acquired a large selection of plastinated specimens representing almost the entirety of gross anatomy. However, we did not completely forego dissection, since, dissection is known to develop manual skills, empathy and teamwork [26, 31]. Moreover, in plastinated specimens, the mobility of the individual structures is lost so that students are unable to remove organs to fully appreciate their neighboring relations.

Several studies indicate that the use of ***computer***-based technologies as part of a combination of teaching tools positively influences students’ acquisition of gross anatomical knowledge [32, 33]. This is corroborated by comments in our survey, such as “…The Complete Anatomy was also very useful, but I would have liked to have more of it integrated in the labs.” and “…Complete Anatomy live in the class it would have been very helpful”. It has been reported that students who employed web-based computer-aided teaching programs performed significantly better in examinations than learners who had never accessed the online content [34]. A comparison of the learning outcomes of students taught anatomy using either the ***Anatomage***® table or dissection did not show any significant difference in student performance, but students were more enthusiastic about learning anatomy on the Anatomage® table and believed that they had learned more [7]. In a cross-sectional study comparing the students’ view on anatomy teaching with either the Anatomage® table or plastinated specimens alone or with a combination of both [8], students were significantly more satisfied with a combination of both teaching strategies, compared to the Anatomage® table or plastinated specimens alone. Stanford et al. (1994) came to a similar conclusion, when evaluating learning outcomes after teaching cardiac anatomy by a combination of dissection and an interactive computer-assisted anatomy instruction program, which yielded better results than each of these teaching methods alone [35]. Another group of studies, however, points to a limited value of computer-animated three-dimensional visualization of anatomical relationships, which also depends heavily on the learners’ spatial abilities [36]. Moreover, the resolution of smaller anatomical structures, e.g., arteries and nerves, is generally rather low so that computer-based anatomy teaching benefits from additional exposure to plastinated and prosected specimens [5]. In addition, if used as a stand-alone educational tool, “Anatomical models outperform their computer-based counterparts for anatomy learning” [12].

Another important tool in our anatomy education is ***Imaging-based*** instruction, using ultrasound as well as 3D computerized MRI and CT images to teach anatomy. We have incorporated ultrasonography into our curriculum at a relatively early stage of student training, because it is a non-invasive and relatively inexpensive way to demonstrate anatomical and pathological structures inside the body, thereby linking basic and clinical education [37] and allowing to learn anatomy in a clinical context. During an introductory session, students are instructed in ultrasound technology, how to handle the ultrasound probes, and how to interpret the sonographic images. The students are then allowed to use the CAE Vimedix® trainers. This way, they can locate healthy organs, their spatial relationships, and pathological changes on the mannequins, thereby mimicking the situation in the living. In addition, they also have access to portable ultrasound devices, which allow them to scan standardized patients. Moreover, the Sectra table, which is also located in the Imaging Center, provides the students with the option to generate a three-dimensional notion of anatomy, based on a variety of normal and pathological CT and MRI images in DICOM format. Patient images can be uploaded to the Sectra educational portal and can then be discussed with the students either in class using the Sectra table or online through the Sectra streaming capabilities.

For the reasons described above, we decided to employ a ***combination*** of educational tools to teach anatomy in our laboratories, including plastic models, plastinated specimens, computer-assisted learning tools, prosected specimens, and imaging modalities. Given students’ different learning styles and the varying preferences for teaching approaches, the wide range of educational tools available at KU’s Department of Anatomy and Cellular Biology is highly appreciated by our students, as illustrated by the results of our survey. Moreover, in times of the COVID-19 epidemics, our broad variety of educational tools was extremely helpful in converting our anatomy laboratory education to online teaching sessions. For example, we have been able to use the Complete Anatomy® program for interactive guided visualization of three-dimensional anatomical relationships. The images and the functionality of the Anatomage® table with the assistance of streaming applications, e.g., Zoom® (©2021, Zoom Video Communications, San Jose, California, USA) or Microsoft Teams® (©2021 Microsoft Corporation, Redmond, Washington, USA), can be delivered online to the student audience. The cameras located within the Dry Laboratories, Instructional Studio, Preparation Room and Imaging Center as well as the associated streaming capabilities allow us to record and broadcast explanations of plastic models, plastinated specimens and radiological images to the students from these venues. In addition, real-time dissections can be made available to the learners.

The CMHS allows the Department of Anatomy and Cellular Biology to continuously pursue novel and developing technologies and programs. We continue to assess new teaching modalities together with the Office of Medical Education, the Office of Academic Affairs, and the student cohort to evaluate if they are more advantageous than our current teaching aids. As part of this initiative, technologies such as Hololens 2 together with programs such as HoloHuman (©2022, GigXR Inc, California, USA) and HoloPatient (©2022, GigXR Inc, California, USA and ©2022, 3D4Medical®, Elsevier, Dublin, Ireland) to conduct possible case-based learning sessions are being explored. Replacement devices, such as EchoNous-KOSMOS handheld ultrasound devices (©2022, EchoNous Inc, Redmond, Washington, USA), which are driven by Artificial Intelligence (AI) to detect anatomical structures and aid in probe placement, are currently being evaluated to expand ultrasound in our anatomical teachings. We are actively looking into the option of introducing 3D printing into our curriculum and are assessing the possibilities of incorporating virtual reality, e.g., EON reality (©2022, EON Reality, Irvine, California, USA) into our anatomy teaching, according to the KU Metaverse initiative.

The limitations of our study lie in the fact that we are a newly established Medical College and therefore can only determine student satisfaction on a small student sample. We do not yet have the capability of performing a longitudinal study. Moreover, we realize that not all institutions may have the financial support available to implement new or upgrade existing facilities.

## Conclusion

After three years of planning and construction, our anatomy facilities are now fully functional. The Medical Students of KU are highly appreciative of the large variety of teaching modalities available, and their performance in standardized anatomical exams exceeded our expectations. These results are of interest to faculty tasked to create modern anatomy teaching facilities based on a combination of prosection, plastinated specimen, cadaver-based dissection, medical imaging, living anatomy and multimedia.

## Data Availability

The original contributions presented in the study are included in the article and any further inquiries can be directed to the corresponding author.
